# Modeling collagen fibril self-assembly from extracellular medium in embryonic tendon

**DOI:** 10.1016/j.bpj.2023.07.001

**Published:** 2023-07-05

**Authors:** Christopher K. Revell, Jeremy A. Herrera, Craig Lawless, Yinhui Lu, Karl E. Kadler, Joan Chang, Oliver E. Jensen

**Affiliations:** 1 Department of Mathematics, University of Manchester, Manchester, United Kingdom; 2 Wellcome Centre for Cell-Matrix Research, Faculty of Biology, Medicine and Health, University of Manchester, Manchester, United Kingdom; 3 Division of Molecular and Cellular Function, Faculty of Biology, Medicine and Health, University of Manchester, Manchester, United Kingdom

## Abstract

Collagen is a key structural component of multicellular organisms and is arranged in a highly organized manner. In structural tissues such as tendons, collagen forms bundles of parallel fibers between cells, which appear within a 24-h window between embryonic day 13.5 (E13.5) and E14.5 during mouse embryonic development. Current models assume that the organized structure of collagen requires direct cellular control, whereby cells actively lay down collagen fibrils from cell surfaces. However, such models appear incompatible with the time and length scales of fibril formation. We propose a phase-transition model to account for the rapid development of ordered fibrils in embryonic tendon, reducing reliance on active cellular processes. We develop phase-field crystal simulations of collagen fibrillogenesis in domains derived from electron micrographs of inter-cellular spaces in embryonic tendon and compare results qualitatively and quantitatively to observed patterns of fibril formation. To test the prediction of this phase-transition model that free protomeric collagen should exist in the inter-cellular spaces before the formation of observable fibrils, we use laser-capture microdissection, coupled with mass spectrometry, which demonstrates steadily increasing free collagen in inter-cellular spaces up to E13.5, followed by a rapid reduction of free collagen that coincides with the appearance of less-soluble collagen fibrils. The model and measurements together provide evidence for extracellular self-assembly of collagen fibrils in embryonic mouse tendon, supporting an additional mechanism for rapid collagen fibril formation during embryonic development.

## Significance

Despite the importance of collagen in the formation and proper functioning of tissues, relatively little is known about how fibril bundles are formed, especially during embryonic development. Current models of cell-directed fibrillogenesis are unlikely to account for the rapid appearance of ordered fibril bundles. We propose a model for collagen fibrillogenesis in embryonic tendon based on self-assembly, supported by mathematical modeling and experimental data.

## Introduction

Collagen is the largest and most abundant protein found in vertebrates (1) and forms the primary structural component of multicellular tissues across multiple scales, ranging from the cornea to tendon and skin. Despite the critical importance of collagen to multicellular life, and its role in conditions such as Ehlers-Danlos syndrome (2) and osteogenesis imperfecta (3), there remain significant gaps in our understanding of the initial development of collagen fibrils (4), especially in the mesoscale regime at scales above that of chemical bonds but below that of organs and tissues.

**Figure 1:**
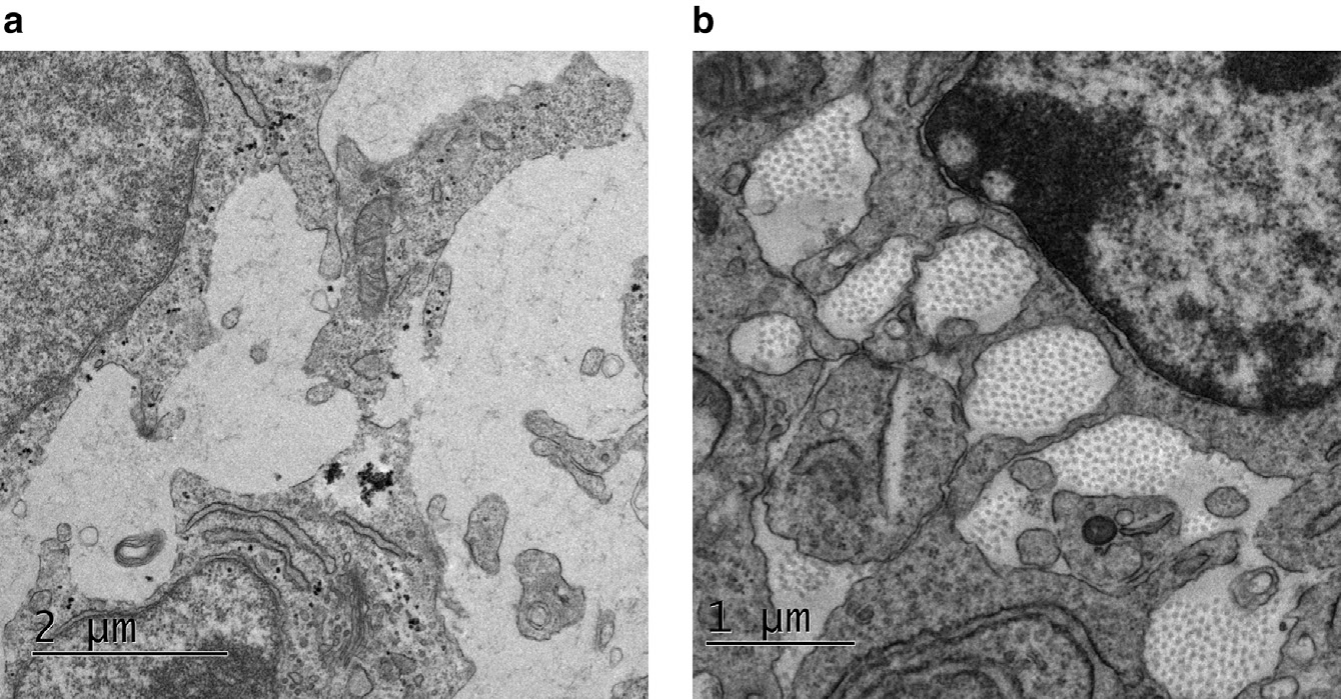
Cross sections of mouse embryonic tail tendons. (*a*) Cross section of mouse embryonic tail tendon at E13.5, showing absence of fibrils in inter-cellular spaces. (*b*) Cross section of mouse embryonic tail tendon at E14.5, demonstrating sudden appearance of fibril lattices in inter-cellular spaces 24 h later. Images are unpublished from study (9). Scale bars: (*a*) 2 μm; (*b*) 1 μm.

Previous cross-section electron microscopy (EM) images of embryonic mouse tail tendon suggest that fibrils appear within a 24-h window between embryonic day 13.5 and 14.5 (E13.5–14.5), forming parallel fibers that appear in cross section as a roughly hexagonal lattice of approximately identical circles (Fig. 1). This lattice develops in inter-cellular spaces, which are themselves extended tubes parallel to the long axis of the tendon (5). The highly ordered nature of fibrils within tendon led to the assumption that cells must exert tight active control over their development, and thus fibrils were hypothesized to be actively laid down only at cell surfaces (6). This requires that cells retain contact with the collagen fibril and grow it in multiple ways: 1) by continuously feeding new collagen protomer at the contact site, which would require the fibrils to move extensively through the inter-cellular space as the fibril grows (7); 2) cells may instead move along the fibril with the growing tip embedded within the cell; or 3) cells remain in situ with several smaller fibrils assembled at cell surfaces, which are subsequently attached together end to end (8). The cell-mediated fibrillogenesis hypothesis is supported by observations of structures known as fibripositors, within which collagen fibrils were shown to be embedded within a cell (9). However, the existence of fibripositors was previously only observed at E14.5 and not E13.5, when fibrils have already extensively occupied the inter-cellular spaces (9). Further, highly ordered collagen fibrils observed at the center of dense fibril bundles (Fig. 1 *b*), where cell surfaces are not in close proximity, also raise the question of how cells may coordinate rapid but precise movements (of either the cell body or multiple fibril ends) that transform the inter-cellular spaces from voids to dense ordered bundles of collagen fibrils, all within 24 h. Here, we explore a mechanism complementing cell-mediated fibrillogenesis by considering the rapid appearance of ordered fibril arrays within a disordered medium as a phase transition or crystallization process (10).

We investigate this hypothesis with a combined experimental and theoretical approach. We simulate self-assembly of fibrils using a phase-field crystal (PFC) model, a standard mathematical description of self-assembly, as outlined in “mathematical modeling.” We perform simulations in irregular domains derived from inter-cellular spaces observed in EM images of mouse tail tendon at the stage of development when fibrils first appear. We compare the results of these simulations to corresponding patterns of fibrils observed directly in these EM images and discuss similarities and differences, in particular noting the density of defects and the presence of voids in fibril packing.

Self-assembly of collagen fibrils is predicated on the hypothesis that protomeric collagen is secreted by cells into the inter-cellular space before the formation of visible collagen fibrils. Crystallization of this extracellular pool of collagen protomers is in contrast to previous suggestions of active assembly of fibrils by cells at cell surfaces (6) but does not preclude the involvement of cell-directed fibrillogenesis in this process. Rather, it may work in tandem to aid rapid growth of fibrils during embryonic development. We validate this hypothesis by experimental observation of the contents of inter-cellular spaces in embryonic tendon, using laser-capture microdissection (LCM) coupled with mass spectrometry (MS) analysis (LCM-MS) (11). The results suggest a steady increase of collagen and proteins involved in collagen fibrillogenesis over time up to E13.5, followed by a sharp reduction of available collagen in the inter-cellular space between the time points where collagen fibrils appear in the tendon tissue. This supports the phase-transition model proposed here, where the available collagen, easy to extract for LCM-MS (12), peaks before the formation of observable fibrils, when protomers are incorporated into less-soluble collagen fibrils in a crystallization process.

## Materials and methods

### Mathematical modeling

#### The PFC model

We hypothesize that fibrils form by crystallization of an existing pool of collagen protomers secreted into the inter-cellular space before the visible appearance of fibrils, with fibril ordering determined by the geometry of the inter-cellular space. We model the extracellular self-assembly of collagen protomers into fibrils using the PFC equation (13,14,15) (Appendix A), a sixth-order nonlinear partial differential equation that has been used extensively to model the emergence of ordered structures from a disordered medium (16), including crystal nucleation in colloidal suspensions (17,18) and even the internal structure of collagen fibrils themselves (19,20). The PFC equation is a coarse-grained model derived from explicit inter-particle interactions (21,22); this coarse-graining ensures that the PFC method is scalable to larger systems but remains grounded in nano-scale physics. However, rather than formally derive a free energy specific to collagen from first principles, we assume a canonical functional form with a minimal number of parameters. To reduce computational cost, our simulations describe evolution in a two-dimensional plane normal to a fibril bundle, neglecting variation along the long axis of inter-cellular spaces. In addition to symmetry along the long axis of inter-cellular spaces (5), we assume that the transverse isotropy of resulting fibril arrays is enhanced by a variety of factors, such as nematic ordering of free collagen protomers (23), geometric confinement (24), or longitudinal mechanical stress (25,26,27), that together promote nematic alignment of fibrils along the axis of a developing tendon. Explicitly modeling 3D alignment using anisotropic forms of the PFC (28,29,30) was considered to be beyond the scope of this study.

#### The phase field

A phase field is a function that divides a domain into two primary states, labeled with different scalar values, in this case crystallized collagen fibril (in which collagen is highly condensed) and extracellular medium (in which collagen protomers exist at relatively low concentration). More precisely, the PFC model describes the evolution of a spatial phase field ϕ(x,t), where *ϕ* varies in the range −1 to 1. The evolving phase field divides the solution domain into regions $\Omega_{\pm }$ for which ϕ(x,t)≈±1 for $x\in \Omega_{\pm }$, with narrow interfaces over which *ϕ* varies with a sharp gradient. We use ϕ(x,t)≈−1 to indicate that fibrillar collagen exists at position $x\in \Omega_{-}$ and time *t*, and ϕ(x,t)≈1 to indicate that protomeric collagen suspended in extracellular medium exists at $x\in \Omega_{+}$. The PFC equation models the phase separation of these two states, and consequently the crystallization of fibrillar collagen from an initial state. The phase field measures density fluctuations, so that the quantity $c=\frac{1}{2}\left(1-\phi \right)$ provides a crude (dimensionless) proxy for molecular concentration: we consider $0\leq c<\frac{1}{2}$ to define regions occupied by protomer (with low molecular density) and regions with $\frac{1}{2}<c<1$ to define fibrillar regions in which collagen is densely aggregated. Phase separation is driven by gradients of free energy; however, the system exhibits glassy dynamics, meaning that multiple metastable states can arise, depending on fine details of the initial conditions in any realization of the model. Given the rapid diffusion of protomeric collagen (31) relative to the timescale of fibril assembly and length scale of the inter-cellular space, we assume a roughly uniform initial *ϕ* field with a small random fluctuation specified by a Gaussian random field. This disordered initial state leads us to take a statistical view of the fibril patterns that emerge.

#### Parameters

Spatial scales of the PFC are set by a parameter $q^{*}$ that is adjusted to fit length scales measured via microscopy, as explained in Appendix A. Additionally, the PFC model has two dimensionless parameters (*r* and $\phi_{0}$) that can be related to the free energy that drives fibril assembly (Appendix A). The parameter *r* measures the destabilizing component of the free energy (relative to a stabilizing component that penalizes density gradients); $\phi_{0}$ measures the mean phase field over the domain (effectively specifying the initial collagen concentration). These parameters regulate the spatial patterns formed by steady-state solutions of the PFC model (13). Within certain ranges, however, predicted fibril patterns are statistically robust with respect to parameter variations. The only additional parameters are a correlation length *λ* and variance $m^{2}$ of the initial state, specified as a Gaussian random field (32); again, predicted patterns show little sensitivity to reasonable variations of these parameters.

#### Computational methods

We applied a split semi-implicit approach to solving the PFC equation (33), using the Julia differential equations library (34,35) to achieve good computational performance (Appendix B). To accommodate potential fibril nucleation at domain boundaries, fibril self-assembly was modeled by solving the PFC equation in irregular domains defined by inter-cellular spaces, extracted from electron microscopy (EM) images of embryonic mouse tail tendons in cross section (Appendix C), allowing the influence of domain shape on fibril patterns to be investigated. To compare fibril patterns in EM images to fibril patterns in simulations, we mapped patterns of spatial defects by using a Delaunay triangulation to identify the number of nearest neighbors of each fibril. We measured deviations from the expected number of neighbors to quantify the irregularities in the packing and to analyze the distribution of fibril neighbor separation and neighbor counts.

We selected values for parameters *λ* and $m^{2}$ based on initial sense-testing, confirming that these parameters did not significantly affect equilibrium states of the PFC equation, selected a time step dt that ensured stability, and ran to a maximal time that ensured a near-equilibrium final state (Appendix D). We then ran many simulations in a wide region of $\left(r,\phi_{0}\right)$-space to investigate the set of possible outcomes from random initial conditions.

### Image analysis

We applied a similar image analysis pipeline to both EM images and simulation results (Appendix E). Starting from a representative set of EM images showing inter-cellular spaces and collagen fibril bundles, we developed a pipeline to manually identify locations of fibrils. Manual selection was preferred over segmentation due to limitations of noisy EM images. We performed a Delaunay triangulation (36) over this set of points for each image (Appendix E). With these Delaunay triangulations, we were able to analyze the patterns of nearest neighbors for each image, including the distribution of spacing between nearest neighbors, and the distribution of neighbor counts for each fibril.

The neighbor count of each fibril within the triangulation reveals defects in the lattice pattern (37). We visualized the neighbor counts of fibrils as a Voronoi tessellation (36), with each Voronoi cell representing one fibril and the color of the cell being clear if the fibril has six neighbors, red if the fibril has five neighbors, or blue if the fibril has seven neighbors. We calculated the defect proportion (*d*) for each image by taking the ratio of the number of fibrils that do not have six neighbors to the total number of fibrils, excluding those on the periphery of the bundle.

### Biological samples

#### Mice

The care and use of all mice in this study were carried out in accordance with the UK Home Office Regulations, UK Animals (Scientific Procedures) Act of 1986 under the Home Office License (PP3720525). Twelve-week old female C57BL/6 mice were time mated with males that were removed after overnight housing. Embryo ages were calculated as day 0 on the morning where females were plugged, and embryos were isolated at ages 12.5 days, 13 days, 13.5 days, 14 days, and 14.5 days (E12.5, E13, E13.5, E14, E14.5). Embryos were randomly sampled for each analysis technique. Embryos were kept in ice-cold PBS and tail tendons were removed at the base. These tail tendons were then fixed with 4% formalin for LCM, or EM fixative (2% glutaraldehyde/100 mM phosphate buffer at pH 7.2).

#### EM

After fixation, the tails were washed in double-distilled H_2_O (${ddH}_{2}$O) for 5 min, repeated three times. The samples were then transferred to 2% osmium (v/v)/1.5% potassium ferrocyanide (w/v) in cacodylate buffer (100 mM, pH 7.2) and further fixed for 1 h, followed by extensive washing in ${ddH}_{2}$O. This was followed by 40 min of incubation in 1% tannic acid (w/v) in 100 mM cacodylate buffer, and then extensive washes in ${ddH}_{2}$O. Samples were then placed in 2% osmium tetroxide for 40min, followed by extensive washes in ${ddH}_{2}$O. Samples were incubated with 1% uranyl acetate (aqueous) at $4^{\circ }$C for at least 16 h, and then washed again in ${ddH}_{2}$O. Samples were then dehydrated in graded ethanol in the following regime: 30%, 50%, 70%, 90% (all v/v in ${ddH}_{2}$O) for 8 min at each step. Samples were then washed 4 times in 100% ethanol, and transferred to pure acetone for 10 min. The samples were then infiltrated in graded series of Agar100Hard in acetone (all v/v) in the following regime: 30% for 1 h, 50% for 1 h, 75% for 16 h, and 100% for 5 h. Samples were then transferred to fresh 100% Agar100Hard in labeled molds and allowed to cure at $60^{\circ }$C for 72 h. Sections (80 nm) were cut and examined using a Tecnai 12 BioTwin electron microscope.

#### Histological staining and imaging

Five-micrometer sections of formalin-fixed and paraffin-embedded (FFPE) mouse tail specimens were H&E-stained by using an automated stainer (Leica XL) at University of Manchester’s Histology Core as previously described (11,38). We used a DMC2900 Leica instrument with Leica Application Suite X software for imaging.

#### LCM of mouse tail tendons

A mouse tail contains four tendon bundles that are uniformly spaced around a central bone (39) (Fig. 4 *a*). The MMI CellCut Laser Microdissection System (Molecular Machines & Industries) was used in combination with their MMI CapLift technology to capture regions of interest within embryonic tail tendon bundles on MMI membrane slides (MMI, 50102) as previously described (11,38). Laser power was set to be between 30% and 40%, cut speed at 50 *μ*m/s, and z drill at 5 *μ*m. In Fig. 4 *b* we show our LCM capacity to cut and capture the four tendons at embryonic time points E12.5 and E14.5. In this study, we capture tail tendons for five time points E12.5, E13.0, E13.5, E14.0, and E14.5 (*n* = 3 mice per time point; a total of 15 samples) with a tissue collection volume of roughly 0.01 ${\text{mm}}^{3}$ per sample. Samples were stored at $4^{\circ }C$ before laser capture and stored at $-80^{\circ }C$ once tendons were collected. The tendons were subject to MS preparation. In short, samples underwent a multistep process to maximize protein yield, including high detergent treatment, heating, and physical disruption, as previously described (11,38).

#### Liquid chromatography coupled tandem MS

The separation was performed on a Thermo RSLC system (Thermo Fisher), as previously described (40). The analytical column was connected to a Thermo Exploris 480 MS system via a Thermo Nanospray Flex ion source via a 20-μm inner diameter (ID) fused silica capillary. The capillary was connected to a fused silica spray tip with an outer diameter of 360 μm, an ID of 20 μm, a tip orifice of 10 μm, and a length of 63.5 mm (New Objective Silica Tip FS360-20-10-N-20-6.35CT) via a butt-to-butt connection in a steel union using a custom-made gold frit (Agar Scientific AGG2440A) to provide the electrical connection. The Nanospray voltage was set at 1900 V and the ion transfer tube temperature set to $275^{\circ }C$.

Data were acquired in a data-dependent manner using a fixed cycle time of 1.5 s, an expected peak width of 15 s, and a default charge state of 2. Full MS data were acquired in positive mode over a scan range of 300 to 1750 *m/z*, with a resolution of $1.5\times 10^{4}$ Full Width at Half Maximum, a normalized Automatic Gain Control target of 300%, and a maximal fill time of 25 ms for a single microscan. Fragmentation data were obtained from signals with a charge state of +2 or +3 and an intensity over 5000 ion/s, and they were dynamically excluded from further analysis for a period of 15 s after a single acquisition within a 10-ppm window. Fragmentation spectra were acquired with a resolution of $1.5\times 10^{4}$ FWHM with a normalized collision energy of 30%, a normalized AGC target of 300%, first mass of 110 *m/z*, and a maximal fill time of 25 ms for a single microscan. All data were collected in profile mode.

## Results

### Simulated fibril pattern formation and maturation

**Figure 2:**
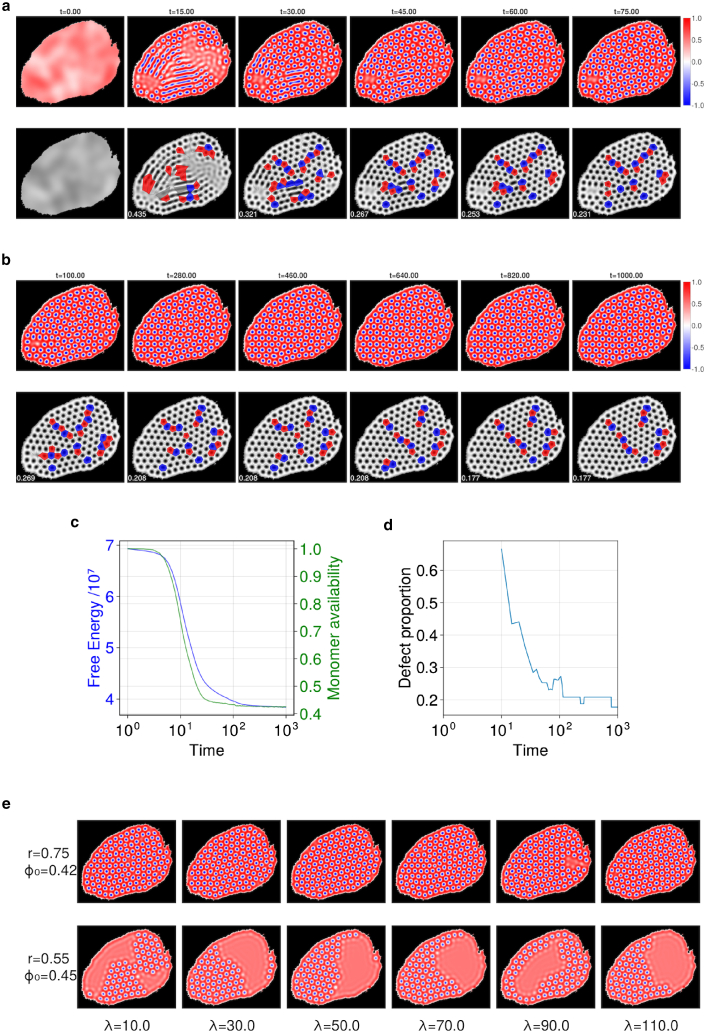
Time evolution of the phase field ϕ(x,t). (*a*) The short-timescale dynamics show rapid emergence of fibrillar structures, with corresponding defect analysis. The top row shows simulation data at six time points, with the color showing the value of *ϕ*; the lower row shows the same data after a defect analysis as described in “image analysis.” A Voronoi tessellation is overlaid on the fibril lattice, with clear cells for fibrils that have six neighbors, red cells for fibrils that have five neighbors, and blue cells for fibrils that have seven neighbors (any other number of neighbors is shown with a gray cell). Each image is 881 nm in the horizontal dimension. (*b*) The long-timescale dynamics show slow coarsening of ordered structures toward the lowest accessible free-energy state. (*c*) Free energy and protomer availability ($c=\frac{1}{2}\left(1-\phi \right)$, integrated over $\Omega_{+}$) against time for the system shown in (*a*) and (*b*). (*d*) Defect proportion against time for the system shown in (*a*) and (*b*). All data for (*a*)–(*d*) were generated with r=0.8, $\phi_{0}=0.4$, and λ=10. (*e*) Example simulation results showing the impact of varying the correlation length *λ* of the initial random field, for two sets of $\left(r,\phi_{0}\right)$ values at t=1000. To see this figure in color, go online.

The short-timescale (Fig. 2 *a*) and long-timescale (Fig. 2 *b*) evolution of the PFC model shows rapid emergence of ordered structures followed by slow coarsening. From a random initial condition (mimicking release of collagen protomer from surrounding cells before the start of the simulation at t=0), the pattern initiates at the boundary of the extracellular domain, propagating inwards toward its center (Fig. 2 *a*). Having filled the domain (in this example), the pattern adjusts slowly over time, with the density of defects in the fibril packing falling slowly as the pattern takes on an increasingly crystalline structure (Fig. 2 *b* and *d*). However, because of the irregularity of the boundary, and because some features of the initial random field are effectively frozen into the pattern, defects are a persistent long-term feature of the fibril array and are present at a sufficiently high density to consider the pattern disordered. Defects appear as disclinations (isolated fibrils with five neighbors, colored red, or with seven neighbors, colored blue in Fig. 2 *a* and *b*), as dislocations (red/blue pairs), and sometimes as linear pleats (or scars) containing an even (or odd) number of red/blue fibrils, which can be considered as grain boundaries (41). Defects are not plotted for fibrils at the periphery of the cluster, leading to fluctuations in the defect proportion among interior fibrils in Fig. 2 *d*, which otherwise falls over time. The free energy driving the evolution falls monotonically as the system reaches a near-equilibrium state (Fig. 2 *c*). Likewise, the availability of collagen protomer falls over time, as protomer is incorporated into fibrils (Fig. 2 *c*). Here we have used $c=\frac{1}{2}\left(1-\phi \right)$ as proxy for collagen density, taking $0\leq c<\frac{1}{2}$ as representative of low-density regions (with collagen in protomeric form, red regions of the color map in Fig. 2 *a*, *b*, and *e*) and $\frac{1}{2}<c\leq 1$ as representative of high-density regions in which collagen aggregates into fibrils (blue regions of color map). The integral of *c* over regions for which $c<\frac{1}{2}$ provides an estimate of the relative availability of protomeric collagen in the extracellular space.

Simulation results are here presented with respect to dimensionless time units, time having been scaled relative to a factor involving free-energy density, collagen mobility, and a length scale. As the free energy is unknown, the model cannot make a priori predictions of the time needed for patterns to initially form. However it does predict that pattern maturation (involving gradual elimination of some defects) takes place over substantially longer timescales (ca. 500 time units) in comparison to the initial pattern formation (ca. 50 time units).

### Localized states and pattern robustness

Fig. 2*e* illustrates the impact of varying the correlation length *λ* of the initial Gaussian random field. For fixed parameters $\left(r,\phi_{0}\right)$, although there is variability between simulations as a result of the random initial conditions, *λ* does not appear to have a significant effect (at a statistical level) on large time configurations. However, the figure illustrates how voids can appear in the fibril array, a feature also evident in Fig. 6 (Appendix D), which illustrates model predictions for a wider parameter sweep in *r* and $\phi_{0}$. Lower values of $\phi_{0}$ and higher values of *r* lead to inter-cellular spaces densely filled with fibril structures, whereas lower *r* and higher $\phi_{0}$ produce regions of dense structures with large gaps of empty space. These can be considered as so-called localized states, a characteristic feature of PFC models (13), whereby fibril patterns can coexist with a homogeneous density field. As each simulation is run from a distinct random initial condition, the shape and location of voids in the pattern are highly variable, although the size of voids is regulated by parameter values. Correspondingly, there is variability in the density and distribution of defects (Fig. 7, Appendix E), although these commonly appear in pairs (as dislocations) or lines (as scars or pleats). In summary, within the range of parameters investigated, the free-energy parameter *r* and the protomer abundance parameter $\phi_{0}$ primarily regulate the appearance of voids in the pattern, whereas stochastic effects determine the precise arrangements of defects (and voids) within the fibril arrays. Consequently, we use below r=0.8 and $\phi_{0}=0.4$ as representative of parameter values leading to complete filling of the extracellular space with fibrils. In the model, only a single length-scale parameter ($q^{*}$) was adjusted to fit model predictions to data.

### Defect analysis

**Figure 3:**
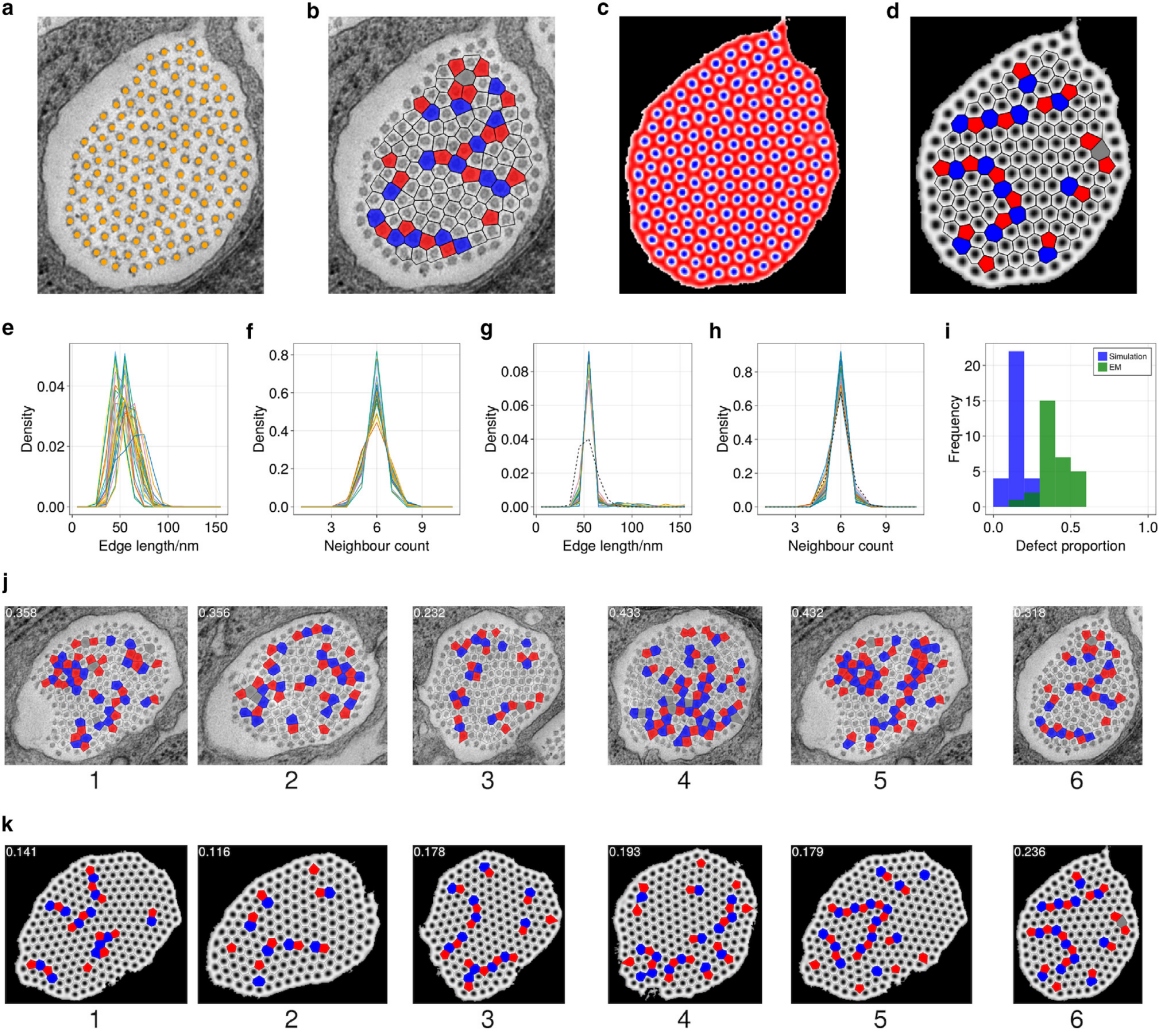
EM images of mouse tail tendon. (*a*) Example of an EM image of an inter-cellular space with fibril locations highlighted by orange dots. (*b*) Corresponding image with defects in the fibril lattice highlighted such that fibrils with five neighbors are colored red and those with seven neighbors are colored blue, excluding peripheral fibrils. (*c*) Final state of a simulation performed in a domain corresponding to the inter-cellular space in (*a*), with parameters r=0.8 and $\phi_{0}=0.4$. (*d*) Voronoi tessellation of result in (*c*) with Voronoi cells colored by neighbor count as in (*b*). (*e*) Normalized distributions of fibril nearest-neighbor spatial separation for all 30 images shown in Fig. 8. (*f*) Normalized distribution of the nearest-neighbor counts for all internal fibrils, for all 30 images shown in Fig. 8. (*g*) Set of normalized distributions of fibril nearest-neighbor-separation distance (edge length) for simulations at 36 points in *r* and $\phi_{0}$ space, run in a domain corresponding to inter-cellular space from image (*a*), with length distribution observed in EM image shown with dotted line. (*h*) Set of normalized distributions of fibril nearest-neighbor count for simulations at 36 points in *r* and $\phi_{0}$ space, run in a domain corresponding to inter-cellular space from image (*a*), with neighbor count distribution observed in EM image shown with dotted line. (*i*) Histogram of defect proportions across 30 tested EM images (green) and 30 simulations (blue) in corresponding inter-cellular spaces with parameters r=0.8 and ϕ=0.4. (*j*) A subset of EM images from Fig. 8 with defects in fibril lattice visualized as in (*b*). (*k*) A subset of simulation results from Fig. 9 run in inter-cellular spaces corresponding to (*j*) with r=0.8 and ϕ=0.4, with defects in fibril lattice visualized as in (*d*). All plots were generated using the Makie.jl plotting library (42). To see this figure in color, go online.

To compare model predictions with observation, we identified the locations of fibrils within EM images of mouse tail tendon (Fig. 3 *a*), identified the patterns of defects (Fig. 3 *b*), and ran simulations in a domain matching that of the image (Fig. 3 *c* and *d*). Although the stochastic nature of the simulation precludes recovery of precise details of the pattern, the configuration of defects is broadly similar. Simulations for the range of $\left(r,\phi_{0}\right)$ values used in Fig. 6 generated predictions of the normalized distributions of nearest-neighbor separation (Fig. 3 *g*) and of fibril neighbor count (Fig. 3 *h*). The predicted edge-length distribution is narrower than that measured in Fig. 3 *a* and *b*, indicating that the model underestimates the geometric disorder in the images, but the neighbor-count distribution is very similar.

To provide a wider comparison of model predictions against observation, 30 EM images showing inter-cellular spaces that contain collagen fibril bundles (Fig. 8, Appendix F), of which six are shown in Fig. 3 *j* and *k*, were analyzed to reveal patterns of defects and to recover normalized distributions of nearest-neighbor separation (Fig. 3 *e*), fibril neighbor count (Fig. 3 *f*), and defect density (Fig. 3 *i*). Although there is some variation between images, there is a peak in median fibril spacing around 60 nm, in line with past fibril diameter measurements (43,44), and a sharp peak in neighbor count at six neighbors, as expected for a primarily hexagonal lattice. Simulations in the same 30 domains (Fig. 9) predicted defect patterns arising at lower density (Fig. 3 *i*), indicating that the model fails to fully capture the topological disorder present in the EM images. In summary, simulations predict patterns of fibril arrangements that share many features of EM images, including localized states leading to voids in the fibril arrays and realistic defect patterns (albeit at a lower density on average than in images), and they provide evidence that rapid fibril formation (reflected by a rapid drop in available protomer) is likely to be followed by slower adjustment of patterns in which the defect density falls slightly.

### Collagens and other proteins essential for collagen synthesis increase over time before the formation of visible fibrils

**Figure 4:**
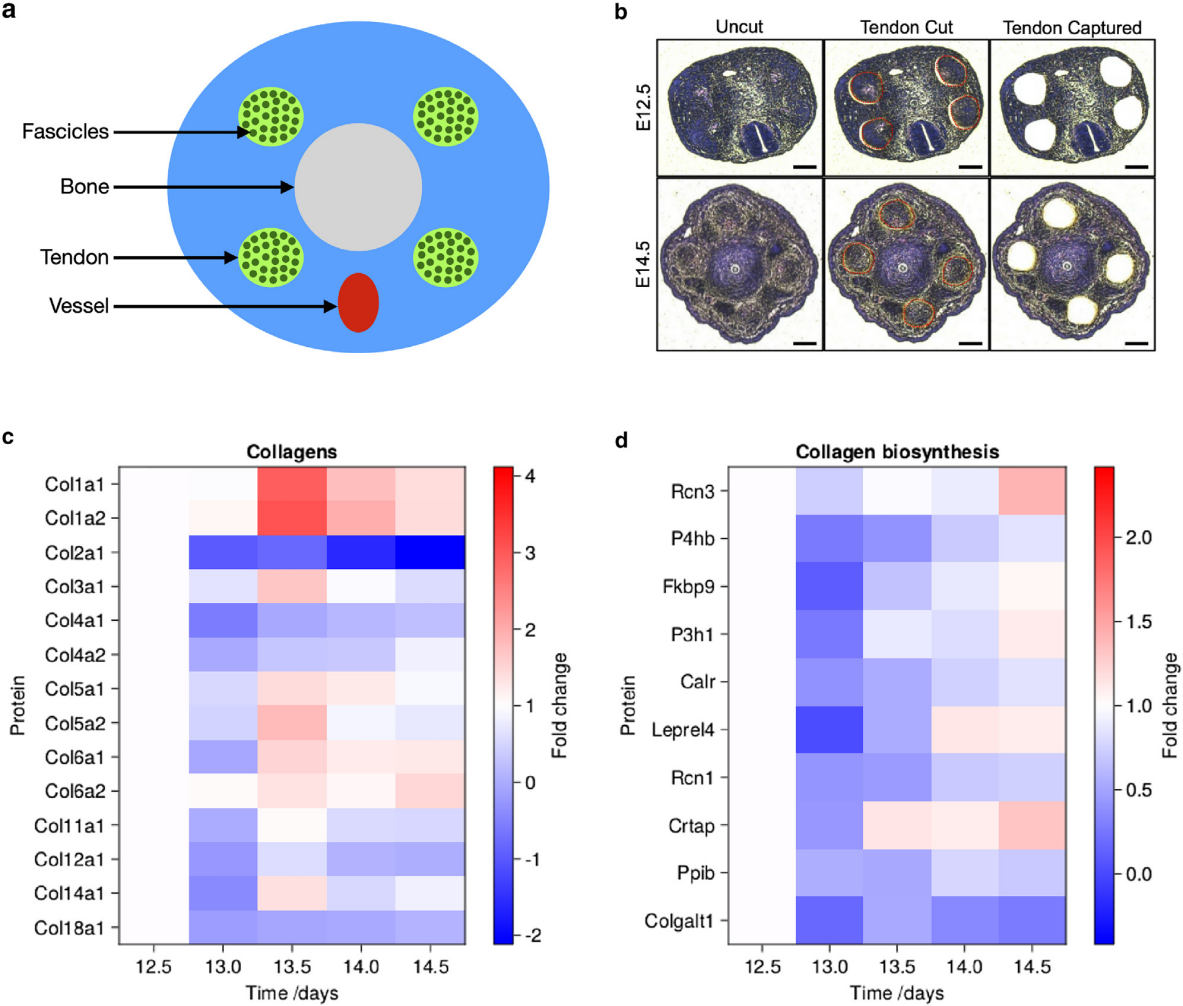
Collagen biosynthesis and collagen proteins change in tail tendons over time. (*a*) A cartoon showing the anatomy of a tail with four tendons, a central bone, and large blood vessel. (*b*) Shown are representative H&E staining images of embryonic mouse tails at embryonic time points E12.5 (upper panels) and E14.5 (lower panels). Tendons were laser-capture microdissected (red dotted circles; middle panels) and captured (right panels) for MS preparation. Scale bars, 100 *μ*m. (*c*) Plot of $\log_{2}$ of fold change in abundance of collagens relative to E12.5. (*d*) Plot of $\log_{2}$ of fold change in abundance of collagen biosynthesis proteins relative to E12.5. To see this figure in color, go online.

To test the model prediction that protomeric collagen should exist in inter-cellular spaces before the formation of visible fibrils, we used MS for protein quantification. We dissected tails from embryos at different stages of development, spanning the ages where collagen fibril formation occurs (E12.5–E14.5) and performed EM analyses to confirm the presence or absence of fibrils along the developmental time series. We observed the emergence of fibrils at E13.5. To identify proteins within regions of collagen fascicles, the major structural component of the tendon (Fig. 4 *a*), both before and after the appearance of collagen bundles, we utilized LCM to specifically isolate the fascicle regions for MS analyses (Fig. 4 *b*).

Our approach quantified 2173 proteins across the five time points. The proteins were compared to the Matrisome project (45) to identify matrix/matrix-associated proteins in our dataset; we identified around 20% of the core Matrisome and 5% of the Matrisome-associated proteins. Sixteen proteins were assigned as collagens, including protein chains making up collagen-I, -III, -V, and -XI, which are central to the formation of the tendon (i.e., collagen fibrils). Further, 30 ECM glycoproteins, seven proteoglycans, 18 ECM regulators, 18 ECM-affiliated proteins, and eight secreted factors were also identified and quantified (Data S1). The abundances of Matrisome proteins across the time series were then normalized to E12.5; proteins where one or more of the time points do not have a value were excluded. Abundances of detected collagens and proteins involved in collagen biosynthesis are presented as heatmaps (Fig. 4 *c* and *d*). The full Matrisome list can be found in Fig. 10 (Appendix G). A heatmap showing the raw protein intensities and a bar chart showing mean protein abundance across all time points can be found in Fig. 11 (Appendix G).

In accordance with EM images showing that collagen bundles start to appear at E13.5, our MS analysis shows that proteins that make up collagen-I protomer (i.e., Col1a1 and Col1a2) significantly increase and peak at E13.5 (Fig. 4 *c*) but then steadily decrease. Other collagens, specifically those responsible for collagen-I fibril nucleation (46) (i.e., Col5a1, Col5a2) showed a similar trend where they peak at E13.5 and then start to decrease in abundance (Fig. 4 *c*). The reduction in collagen-I molecules may be due to the incorporation of soluble collagen-I protomers (either homotrimeric Col1a1 or heterotrimeric Col1a1/Col1a2) into fibrils, a process that makes collagen harder to extract for detection by MS (12). Proteoglycans known to be involved in post-natal tendon development, such as decorin, biglycan, and lumican, were also detected (47,48). Interestingly, many proteins involved in collagen biosynthesis peaked at E14.0 and E14.5, despite a drop in soluble collagen-I levels (Fig. 4 *d*). For instance, P3H1, P4Ha1, P4ha2, P4hb, Ppib, Serpinh1, Rcn3, and Crtap are all critical for collagen biosynthesis (49,50,51,52,53). This is supportive of the interpretation that collagen-I protomers are still being produced but deposited in an insoluble manner (i.e., within a collagen fibril). Taken together, we interpret the MS data to be supportive of the PFC model, where collagen bundles rapidly appear with a concurrent sharp decrease in free collagen protomers.

## Discussion

The PFC model that we have implemented here represents a substantial simplification of collagen biochemistry. However, subject to a number of assumptions, reviewed below, it provides a computationally tractable tool with a small number of free parameters that allows us to investigate the spatiotemporal development of fibrils in inter-cellular spaces within embryonic tendon.

A strong assumption in our approach is that fully resolved molecular dynamics in three spatial dimensions, accounting for fine details of protomer structure, will evolve macroscopically with many of the features that are captured by the relatively inexpensive coarse-grained 2D PFC model (Eq. 1a; Appendix A). Pending the outcome of 3D studies, we can evaluate the evidence supporting this proposition. Imaging of fibril organization in 3D shows a high degree of anisotropy (43) with fibril patterns showing very limited variation along the axis of the tendon. Three factors may be responsible for this. First, protomers are long, thin, and reasonably stiff rods, with a 300:1 aspect ratio and a persistence length roughly the same order of magnitude as their length (54,55). At high concentrations, they can be expected to aggregate in a nematic liquid-crystalline phase, with rods strongly aligned with their neighbors, as has been observed within intracellular vesicles (23,56,57). Second, the protomers occupy long, slender, inter-cellular channels; this geometric confinement can be expected to promote coherent organization of the protomer in a liquid crystal phase (24). Third, mechanical loading along the axis of the nascent tendon can be expected to induce stresses on the protomers that promote coherent alignment and subsequent aggregation (25,26). Together, these factors mitigate against the formation of an isotropic gel, as occurs in vitro (58), but they support use of a simpler 2D model, addressing evolution in the plane normal to the tail tendon, that captures the aggregation of protomers in inter-cellular spaces.

This model is agnostic regarding the initial nucleating trigger, as well as active cell involvement, for fibril formation in the embryonic tendon. Pattern formation in the PFC model arises from any initial fluctuation in *ϕ* across the domain, but the equilibrium results appear independent of whether this fluctuation is localized or distributed throughout the domain. We hypothesize that factors such as biochemical interaction with other species such as collagen-V (46), geometric confinement leading to nematic ordering (24), axial strain by newly forming muscles, or reaching some threshold concentration of collagen protomers are possible nucleation candidates. In this current study, we observed the emergence of fibrils at E13.5, which filled the inter-cellular space by E14. From a biological point of view, factors preventing premature initiation of fibrillogenesis may be several-fold. Collagen-I fibrillogenesis in vitro requires a certain threshold of collagen-I molecules, as well as the presence of nucleators such as collagen-V and collagen-XI (46). Other factors may include a change in pH in the microenvironment (59), cleavage of the propeptide region (60), or the presence of an as-yet unidentified inhibitory molecule against fibrillogenesis. In our data, detection of the known nucleating collagens (collagen-V, collagen-XI) followed the trend of collagen-I, supporting the hypothesis that fibril emergence led to a decrease of free collagen-I protomers. We did not detect the known enzymes for cleavage of the propeptides in procollagen-I (i.e., BMP1, ADAMTS2) and thus cannot draw conclusions on whether a synchronized removal of propeptide regions contributed to the rapid appearance of fibrils. Nonetheless, regardless of what mechanisms are employed by cells to control fibril initiation, the current model does not preclude their existence. Previously, we identified fibripositors in mouse embryonic tendon that supported a cell-directed fibrillogenesis model, where their presence was detected at E14.5 and not at E13.5 (9). In the present study, we observed the emergence of fibrils in E13.5, without the presence of obvious fibripositors; the appearance of fibripositors is only detected from E14 onward (data not shown). From this, we inferred that cell-fibril contacts are less frequent in the initial phase of fibril appearance, suggesting that our alternative phase-transition model provides a key mechanism that mediates rapid fibril growth. This is also suggestive of a two-step embryonic tendon development; however, to further test this hypothesis is beyond the scope of this study.

Fig. 4 provides experimental evidence of protomer abundance during fibril formation. Here, 3.4% of total proteins detected were Matrisome or Matrisome associated, whereas other reports on mouse lungs reported 5.2% (12). The coverage of Matrisome or Matrisome-associated proteins is highly variable, which is influenced by sample preparation method and, importantly, tissue type and age. For example, during embryogenesis, the majority of the tissue is occupied by cells, a phenomenon that is reversed in adults. Regardless, our data demonstrate that there is a steady accumulation of protomeric collagen into the inter-cellular space from E12.5 up until E13.5. The PFC model simulates the subsequent aggregation of this collagen into fibrils. Although the PFC model incorporates randomness in the initial distribution of protomer, it does not directly simulate stochastic effects in the subsequent dynamics. The model predicts a rapid initial phase of aggregation (Fig. 2 *c*) in which the free energy and protomer availability drop rapidly, consistent with Fig. 4 *c*, followed by a slower phase in which the fibril patterns reorganize slowly to lower-energy states with fewer defects (Fig. 2 *d*). Our simulations illustrate the dynamic nature of fibril patterns, highlighting the fact that EM images represent snapshots of an evolving system. Defects are an intrinsic hallmark of fibril patterns; they are present because perfectly crystalline patterns are incompatible with the irregular shape of domain boundaries and because of inherent environmental disorder. Our simulations aimed to reproduce domain boundaries accurately (in 2D, at least; we did not account for axial variations in shape that might have a long-range influence). The noise in our model (incorporated through the initial condition, implemented as a Gaussian random field) highlights the importance of interpreting fibril (and defect) patterns in a statistical sense, because individual realizations of the model show variability in the organization of individual fibrils. Although some simulations show encouraging agreement (Fig. 3 a–d), overall, the density of defects predicted by the model underpredicts the density of defects measured in EM images (Fig. 3 *i*–*k*). Recognizing that defect density varies with time, we attribute the difference primarily to 3D effects, to elevated levels of spatial heterogeneity, and to possible cross-linking of non-equilibrium fibril patterns not captured by the model.

**Figure 6:**
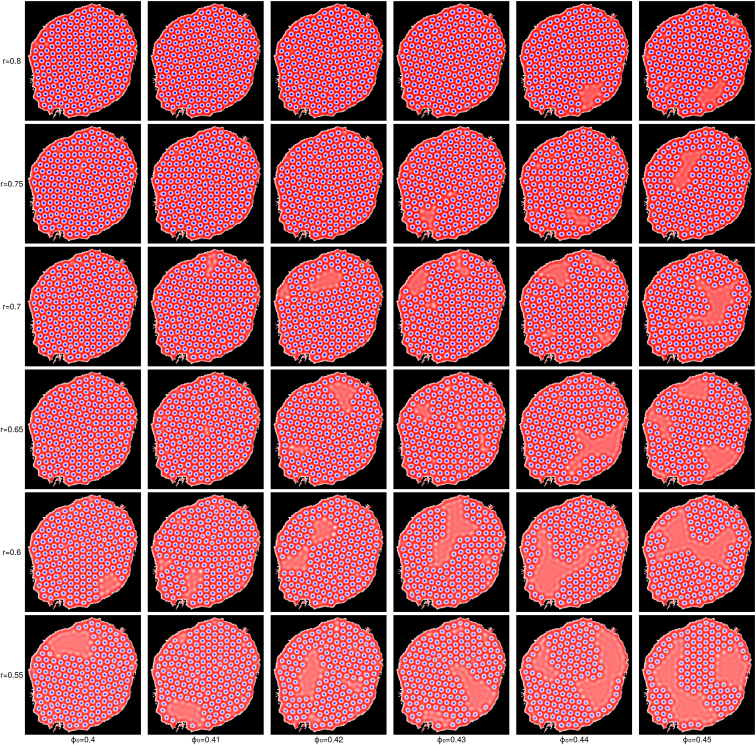
Phase space in *r* and $\phi_{0}$ of PFC solutions in the same solution domain at t=1000. Other parameters are given in Table 1. To see this figure in color, go online.

**Figure 7:**
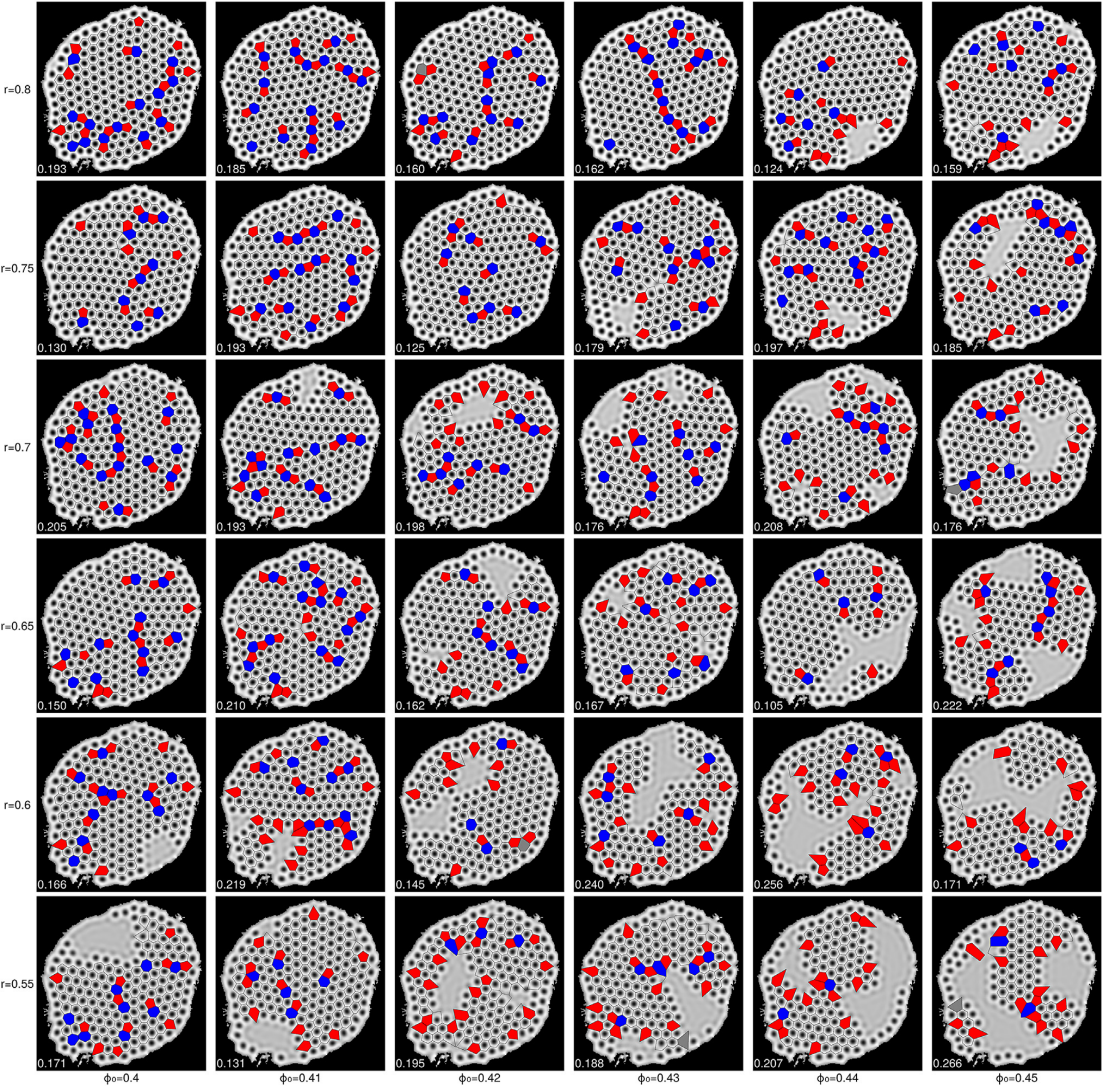
Phase space in *r* and $\phi_{0}$ as in Fig. 6 with Voronoi tessellation over cells and defects in neighbor count highlighted. Voronoi cells above a threshold size are excluded to avoid edge effects from empty spaces in the solution. Fibrils with five neighbors are colored red, fibrils with seven neighbors are colored blue. The number in each image is the defect proportion *d*. To see this figure in color, go online.

**Figure 8:**
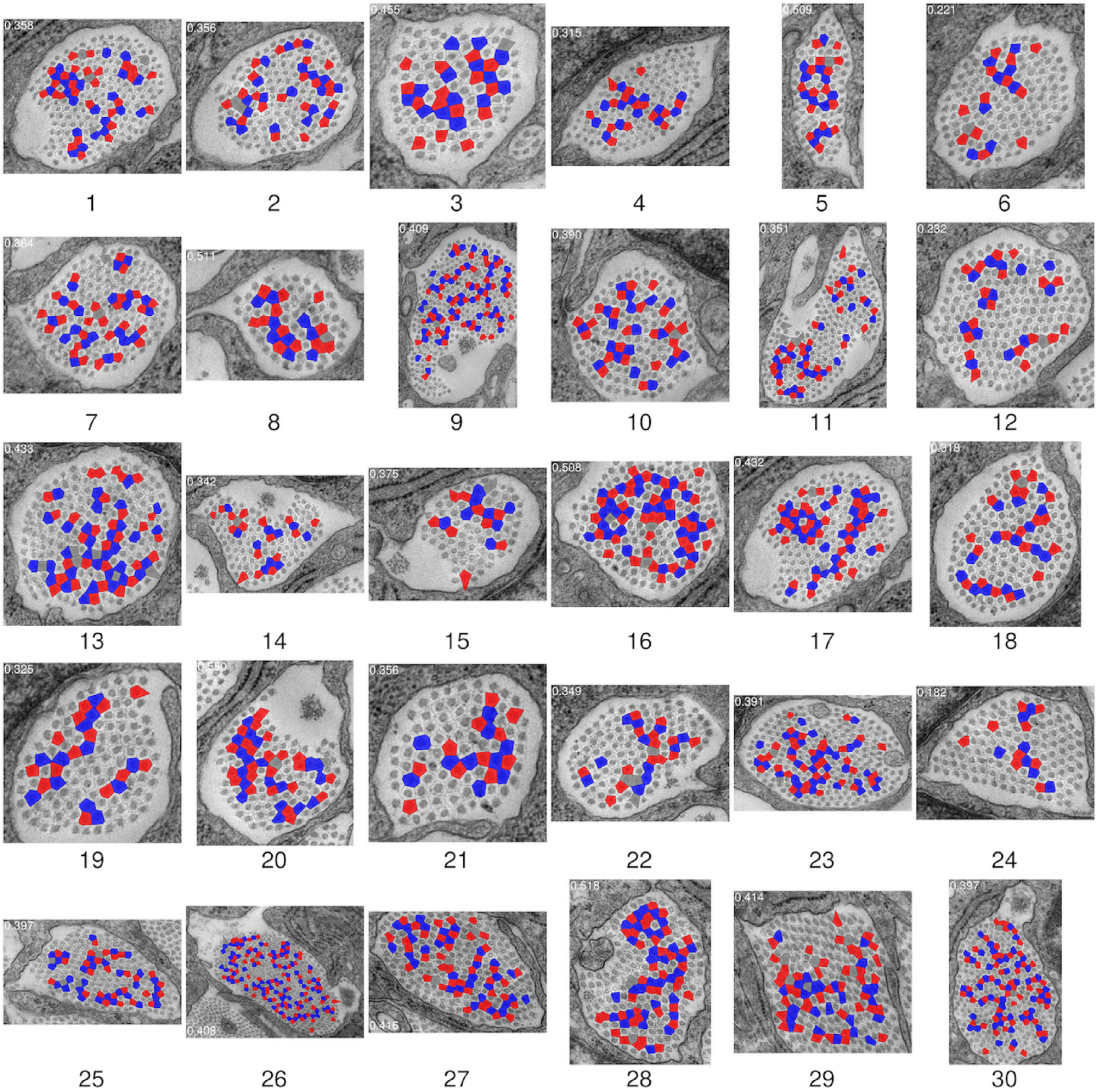
Voronoi tessellation over fibril locations, with Voronoi cells colored by fibril neighbor count. Fibrils with six neighbors are represented by clear cells, five neighbors by red cells, and seven neighbors by blue cells; colored cells therefore indicate the locations of defects in the fibril lattice. The proportion of cells that are defects is recorded in each panel. The mean defect proportion across 30 examples is 0.386. To see this figure in color, go online.

**Figure 9:**
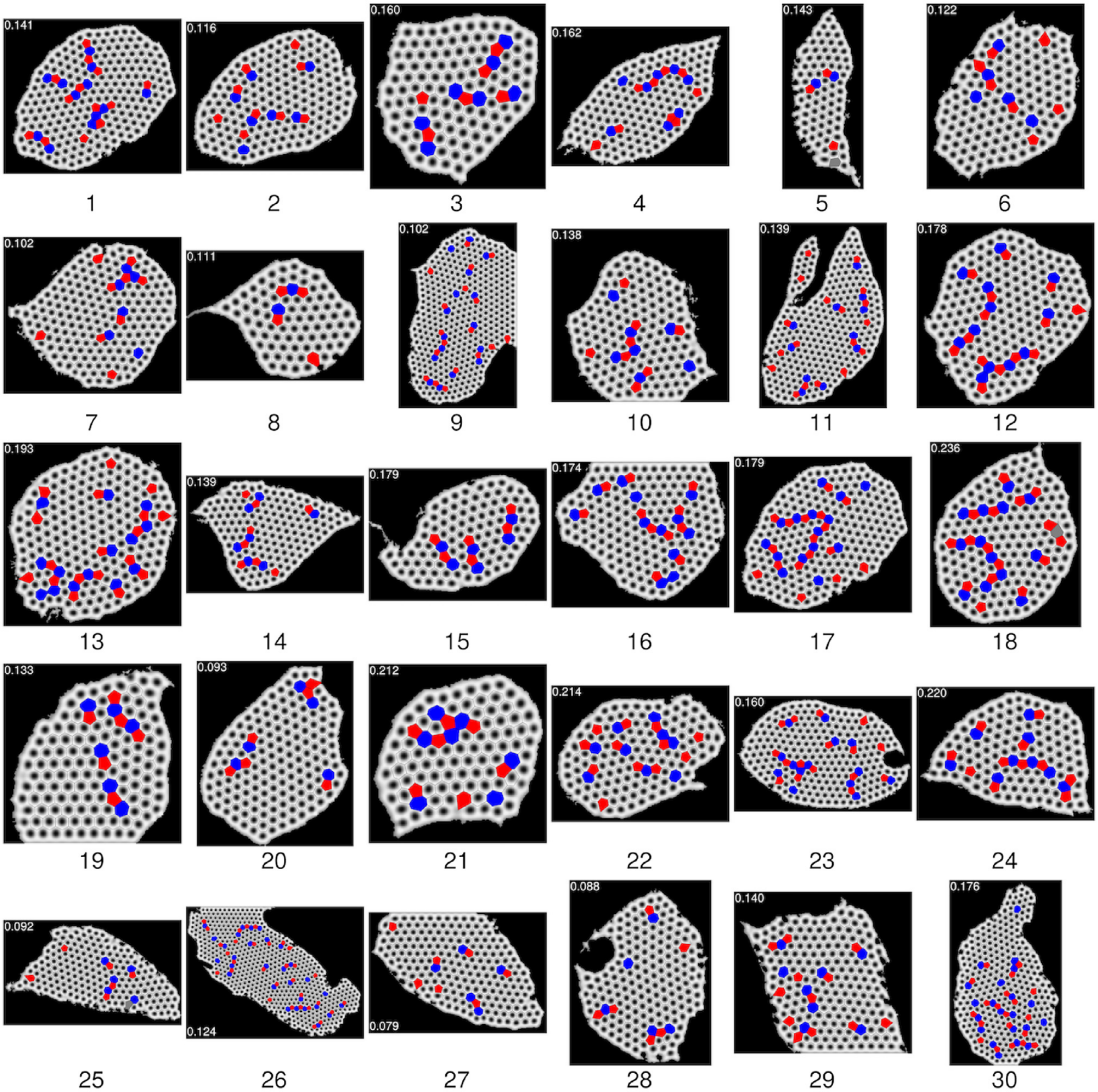
Examples of simulation results for all masks at r=0.8, $\phi_{0}=0.4$, and other parameters as in Table 1. The defect proportion in each image is recorded in the corner of the corresponding panel. The mean defect proportion across these runs is 0.148. To see this figure in color, go online.

**Figure 10:**
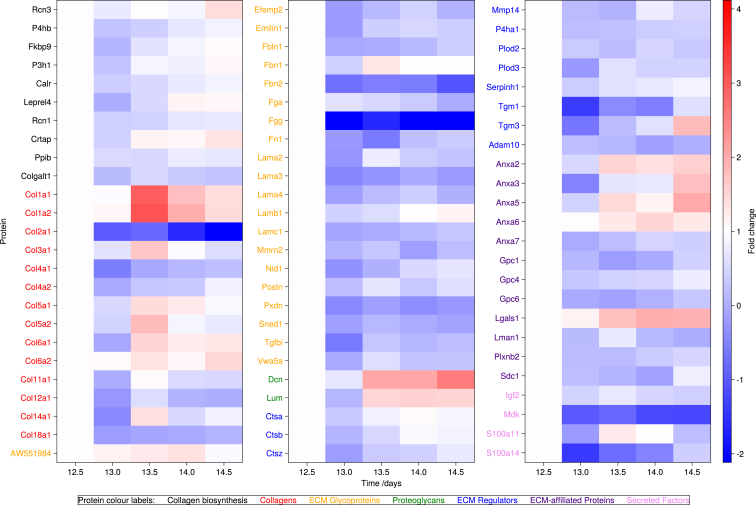
Full heatmap of all relevant protein expression levels over time. Proteins are categorized by function, with different y axis label colors indicating protein categories. All abundance values are given as $\log_{2}$ of fold change, normalized against E12.5, with a white cell indicating no change. To see this figure in color, go online.

**Figure 11:**
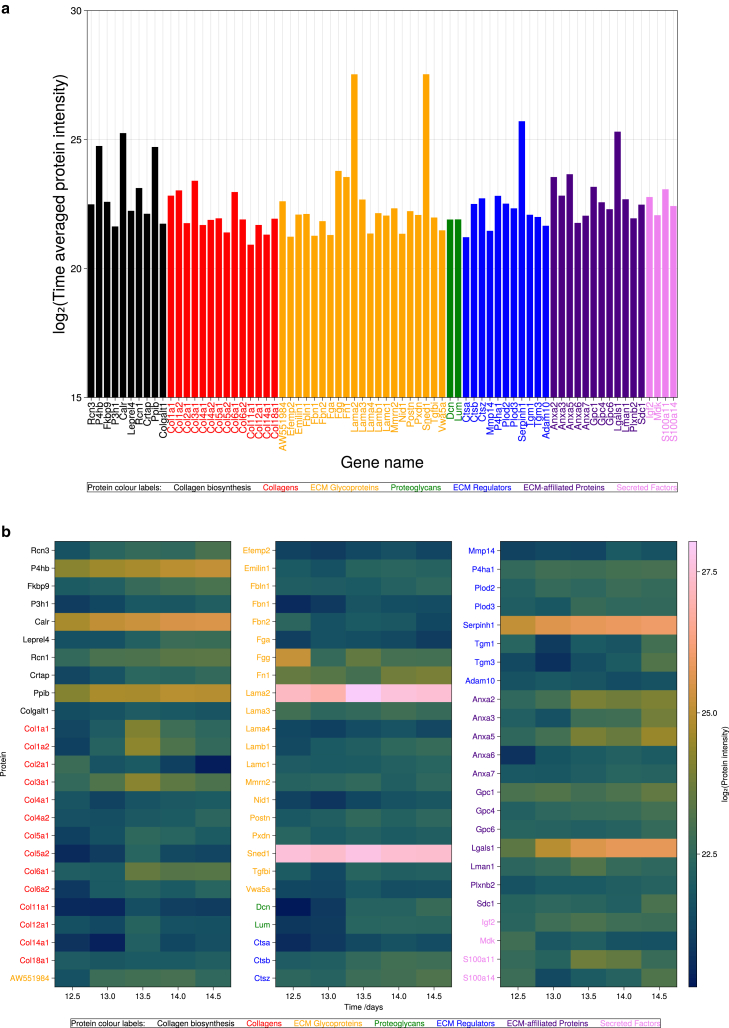
Protein abundance. (*a*) Bar chart showing mean protein abundance across all time points. (*b*) Heatmap showing $\log_{2}$ of protein abundance at all time points. To see this figure in color, go online.

The model does not have sufficient degrees of freedom to capture the detailed biochemistry of collagen protomers, including their chirality and specific cross-linkers or molecules controlling fibril diameter (such as lysyl oxidases or decorin) that regulate self-assembly into fibrils (61). Instead, these effects are aggregated into the free-energy parameter *r* and the initial mean phase field $\phi_{0}$. Although there is considerable uncertainty in the true values of these parameters, fibril formation is predicted when the parameters occupy a range of values (Fig. 6), indicating the robustness of the proposed mechanism. The most striking effect of parameter variation within this allowable range is the appearance of voids in the fibril pattern (for smaller values of *r* and larger values of $\left|\phi_{0}\right|$, see also Fig. 2 *e*). These reflect the fibril-free voids revealed in many EM images within inter-cellular spaces (Fig. 8). Additional secretion of protomeric collagen into the inter-cellular spaces during fibril crystallization, which may be supposed to correspond to a reduction in mean *ϕ* across the domain, may push the system from an equilibrium with voids toward a more uniform fibril lattice, as illustrated in Fig. 6. Although 3D effects are likely to be implicated in void formation in some instances, our model provides an additional potential mechanism, namely as a form of so-called localized state (13) in which fibrils can coexist with regions containing unaggregated protomer. Our simulations closely mimic fibril patterns seen in biological samples and are supported by biochemistry interpreted through our time-series MS data; this is indicative of the feasibility of our model occurring in in vivo systems, albeit in specific scenarios such as embryonic development.

Defects and voids together have implications for the mechanical properties and function of a tendon. Assembly of fibrils within a highly confined environment can be expected to generate internal (residual) stresses through molecular reorganization (62,63) and as a result of geometric frustration (64). Slow reorganization of the fibrils, evident in Fig. 2 *b*, is a form of plastic deformation that allows some stress relaxation. Likewise, under mechanical loading, the internal structure of a newly formed tendon can be expected to reorganize via migration and interaction of defects, through a form of annealing, allowing adaptation of growing tendon to loaded conditions. Although our model describes the rapid initial formation of fibrils via aggregation, it is likely complementary to cell-mediated and cell-controlled fibrillogenesis during this process; in particular, fibripositors (9) are likely to play an important role over long periods, not only in laying down fibrils but also potentially guiding the orientation of fibril arrays via mechanical loading of individual fibrils (27). Further work is required to understand how the strongly anisotropic organization of inter-cellular spaces ensures coherent patterning of fibrils along the axis of the tendon in 3D and mechanisms by which fibril patterns evolve as the embryo matures.

In conclusion, simulations with the PFC model, combined with data obtained by LCM and MS revealing a rapid rise and fall in the abundance of specific inter-cellular collagen protomers, together provide evidence that embryonic tendon fibril formation occurs as a rapid self-assembly process.

## Data availability

The mass spectrometry proteomics data have been deposited to the ProteomeXchange Consortium via the PRIDE (65) partner repository with the dataset identifier PXD043811. All simulation and data processing code is available from GitHub (66).

## Author contributions

O.E.J. and K.E.K. conceived the project. C.K.R. and O.E.J. conceived and developed the mathematical model and image processing. K.E.K. and J.C. supervised experiments. J.C., J.A.H., and Y.L. performed experiments. C.L. performed MS data processing. J.C. and J.A.H. interpreted biological data. O.E.J., C.K.R., J.C., and J.A.H. wrote the manuscript.

## Figures and Tables

**Figure 5:**
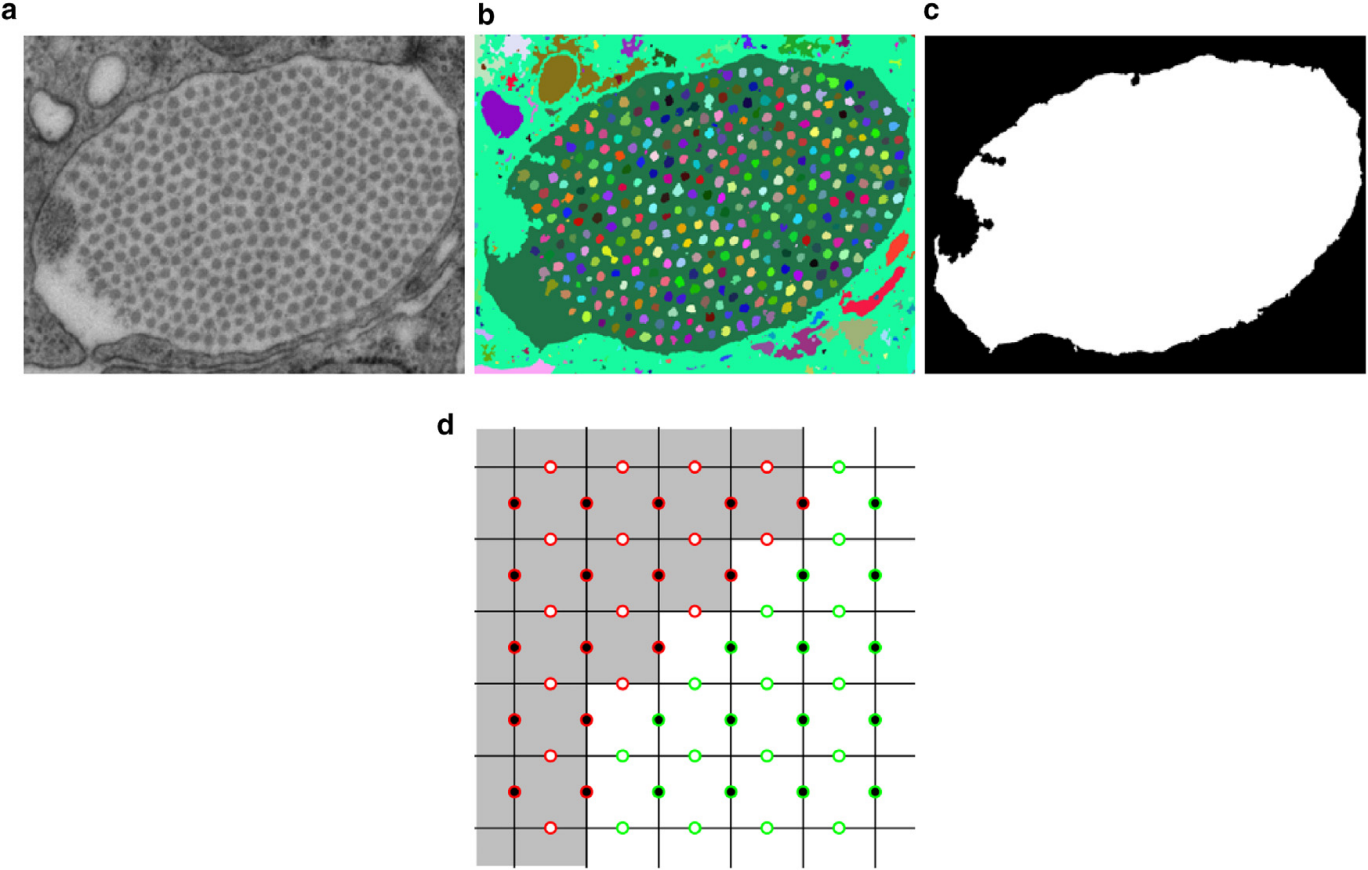
Image processing to define solution domains. (*a–c*) Example pipeline showing the progression from an EM image of embryonic tail tendon of *y*-dimension size *N* and *x*-dimension size *M* (*a*) to a binarized and segmented image (*b*) and finally a mask of inter-cellular and cellular spaces (*c*) obtained by pruning smaller segments. (*d*) Diagram demonstrating how diffusivity matrices are constructed from binarized image mask. Circles represent diffusivity values between pixels in the binarized image. Black filled circles are x-dimension diffusivity values; white-filled circles are y-dimension diffusivity values. Pixel colors represent corresponding black (0) and white (1) components in the binarized image mask. Red circles show where diffusivity values are set to zero; green circles show where diffusivity values are set to 1. To see this figure in color, go online.

**Table 1:** Parameters used to produce results

Parameter	Value
$\phi_{0}$	0.4–0.45
*R*	0.5–0.8
*A*	2.0
dt	0.1
*M*	0.1
tMax	1000
*Λ*	10

**Table 2:** Table specifying neighbor number counts and defect proportions (*d*) for images and corresponding simulations

K	EM images						Simulations					
	4	5	6	7	8	*d*	4	5	6	7	8	*d*
1	1	27	95	22	3	0.358	0	13	152	12	0	0.141
2	1	21	76	19	1	0.356	0	9	122	7	0	0.116
3	1	15	36	14	0	0.455	0	6	68	7	0	0.160
4	0	15	61	13	0	0.315	0	9	88	8	0	0.162
5	0	15	27	12	1	0.509	1	3	36	2	0	0.143
6	0	12	74	8	1	0.221	0	7	86	5	0	0.122
7	1	26	84	20	1	0.364	0	9	132	6	0	0.102
8	0	13	22	9	1	0.511	0	4	48	2	0	0.111
9	2	49	143	46	2	0.409	0	18	299	16	0	0.102
10	0	22	64	18	1	0.390	0	8	94	7	0	0.138
11	4	30	109	24	1	0.351	0	18	179	11	0	0.139
12	0	18	106	13	1	0.232	0	14	120	12	0	0.178
13	5	30	89	32	1	0.433	0	18	134	14	0	0.193
14	0	16	52	10	1	0.342	0	8	93	7	0	0.139
15	1	9	30	8	0	0.375	0	6	55	6	0	0.179
16	1	34	63	28	2	0.508	0	11	109	12	0	0.174
17	1	32	83	29	1	0.432	0	17	147	15	0	0.179
18	0	19	75	15	1	0.318	0	16	97	13	1	0.236
19	0	14	54	12	0	0.325	0	6	78	6	0	0.133
20	1	23	36	19	1	0.550	0	6	107	5	0	0.093
21	0	11	38	10	0	0.356	0	7	52	7	0	0.212
22	2	14	54	11	2	0.349	0	12	77	9	0	0.214
23	1	35	109	33	1	0.391	0	16	157	14	0	0.160
24	0	9	72	7	0	0.182	0	11	71	9	0	0.220
25	3	26	91	30	1	0.397	1	6	118	5	0	0.092
26	6	77	235	78	1	0.408	0	31	425	29	0	0.124
27	0	40	104	31	3	0.416	0	7	140	5	0	0.079
28	1	38	68	32	2	0.518	0	6	114	5	0	0.088
29	1	41	102	25	5	0.414	0	12	123	8	0	0.140
30	0	56	161	48	2	0.397	0	20	192	21	0	0.176

## Appendix A: The PFC model

The PFC model in two spatial dimensions describes the evolution of an order-parameter field $\phi^{*}\left(x^{*},t^{*}\right)$ down gradients of free energy; here, stars denote dimensional quantities. We adopt a canonical free energy with density $\frac{1}{2}C^{*}\left(q^{*4}\phi^{*}-2q^{*2}{\left|\nabla^{*}\phi^{*}\right|}^{2}+{\left(\nabla^{*2}\phi^{*}\right)}^{2}\right)-\frac{1}{2}r^{*}\phi^{*2}+\frac{1}{4}\lambda^{*}\phi^{*4}$, where $C^{*}$, $q^{*}$, $r^{*}$, and $\lambda^{*}$ are positive constants. The corresponding chemical potential density (the first variation of the free-energy density, measured in units of energy $k_{B}T$ per area) is $\mu^{*}=C^{*}L^{*2}\phi^{*}-r^{*}\phi^{*}+\lambda^{*}\phi^{*3}$, where $L^{*}\equiv q^{*2}+\nabla^{*2}$. The rate of evolution is regulated by a baseline mobility $\alpha_{0}^{*}$, which can be expressed as $D^{*}/k_{B}T$ for some diffusion coefficient $D^{*}$. To express the model in nondimensional terms (minimizing the number of parameters), we measure lengths in terms of $1/q^{*}$, so that $\nabla^{*}=q^{*}\nabla$, and time in terms of $1/\left(\alpha_{0}^{*}C^{*}q^{*6}\right)$; we define $r=r^{*}/\left(C^{*}q^{*4}\right)$ (which compares the long-wave-destabilizing contribution to the free energy $\frac{1}{2}r^{*}\phi^{*2}$, with the term setting the primary length scale of the pattern $\frac{1}{2}C^{*}q^{*4}\phi^{*2}$) and rescale $\phi^{*}$ using $\phi^{*}\left(x^{*}\right)={\left(C^{*}q^{*4}/\lambda^{*}\right)}^{1/2}\phi \left(x\right)$.

We use the resulting dimensionless phase field ϕ(x,t) to distinguish regions occupied by extracellular collagen protomer, where ϕ≈1, from regions in which collagen assembles into fibrils, where ϕ≈−1. The free energy is symmetric under the transformation ϕ→−ϕ, but we choose a parameter regime where self-assembled collagen forms arrays of spots. The evolution of *ϕ* under the PFC model (13) can then be written as

(1a) $$\frac{\partial \phi \left(x,t\right)}{\partial t}=\nabla \cdot \left(\alpha \nabla \left({\left(\nabla^{2}+1\right)}^{2}\phi -r\phi +\phi^{3}\right)\right)\text{,}$$

(1b) $$\phi \left(x,0\right)=mG\left(x,\lambda \right)+\phi_{0}\text{.}$$

The time evolution of *ϕ* is thus controlled by the scalar parameter *r*, specifying the destabilizing component of the free energy, the mean value of the phase field over the domain $\phi_{0}$, a variance $m^{2}$, and a correlation length *λ*. We use the mobility field α(x) to define accessible (extracellular) and inaccessible (intracellular) regions within solution domains, such that α=1 in the extracellular region and α=0 in the intracellular region. The spatial domain is discussed further in Appendix C. The PFC equation (Eq. 1a) under periodic boundary conditions is conservative, ensuring that the mean *ϕ* over the solution domain is conserved during the dynamics. The initial condition (Eq. 1b) specifies the phase field using a differentiable Gaussian random field G(x,λ) (32) having square-exponential covariance, unit variance, mean 0, and correlation length *λ*. Thus although Eq. 1a is deterministic, disorder enters via the initial condition at a level determined by the amplitude *m*.

The dispersion relation associated with Eq. 1a for small-amplitude perturbations around $\phi =\phi_{0}$, having growth rate *σ* and wavenumber *k*, is (with α=1 (13))

(2) $$\sigma =k^{2}\left(r-3\phi_{0}^{2}-{\left(1-k^{2}\right)}^{2}\right)\text{.}$$

At $r=3\phi_{0}^{2}$, this reveals neutrally stable modes with wavenumbers zero and unity respectively. Elevating *r* above $3\phi_{0}^{2}$ destabilizes the long-wave mode and gives rise to the existence of localized states in which patterned and unpatterned regions may coexist (13). We hypothesize that this model can thereby explain some features of observations of inter-cellular spaces that are partially filled with fibrils.

Writing the mean nearest-neighbor separation of fibrils in each EM image (Fig. 3 *g*) as $\ell^{*}$, and anticipating from (2) that the (dimensional) wavelength of localized states in the PFC should be $\approx 2\pi /q^{*}$ (corresponding to k=1), we set $q^{*}=\frac{2\pi }{\ell^{*}}$. The domain width of an EM image $L^{*}$ then appears as the parameter $L=q^{*}L^{*}$ in simulations.

We focus (mostly) on equilibrium patterns of fibrils and restrict attention to regimes in $\left(r,\phi_{0}\right)$ parameter space where patterns of spots appear, seeking to understand the credibility of the model in predicting fibril patterns (Fig. 6). We evaluate the robustness of predictions against variations in *r*, $\phi_{0}$ variations in *m* and *λ*.

## Appendix B: Numerical method

The PFC model (Eq. 1) is sixth order in space and, therefore, challenging to solve computationally over long time intervals. We applied a semi-implicit splitting scheme (33) that introduces a splitting parameter *a* and splits the partial differential equation (PDE) into two components that together sum to the original PDE. These components are a linear operator M, which is treated implicitly, and a nonlinear component F, which is treated explicitly, as

(3) $$\frac{\partial \phi }{\partial t}=M\phi +F\left(\phi \right)\;\text{where}\;M=\nabla \cdot \alpha \nabla \left(1-r+a+\nabla^{4}\right),F\left(\phi \right)=\nabla \cdot \alpha \nabla \left(\phi^{3}-a\phi +2\nabla^{2}\phi \right)\text{.}$$

The two terms containing the splitting factor *a* together sum to 0. As recommended in past literature (33), we use a value of a=2.

We construct M and F from second-order central-difference Laplacians using a Von Neumann neighborhood five-point stencil. Due to the spatially varying diffusivity, we must consider the outermost gradient and divergence differential operators (∇·α∇) separately rather than constructing a Laplacian. The *α* field is generated from EM images as described in Appendix C. We developed numerical methods using the Julia programming language (34). In particular, integration was handled by the Julia DifferentialEquations.jl library (35), using the SplitODEProblem functionality. We used a second-order exponential Runge-Kutta scheme with fixed time stepping and with a Krylov approximation/operator caching and Krylov subspace of size 50. After discretization, the mean *ϕ* value across the domain was found to be conserved over time to nine significant figures.

The initial conditions were generated with the Julia GaussianRandomFields.jl package, passing arguments for unit variance and zero mean. Given this Gaussian random field, we set the initial condition to be ϕ(x,t=0)=0 for x within the intracellular space (as defined in Appendix C), and $\phi \left(x,t=0\right)=mG\left(x,\lambda \right)+\phi_{0}-e$ for x within the inter-cellular space. *m* is an amplitude, here set to 0.1, and *e* is the calculated mean of the original Gaussian random field over the inter-cellular domain, such that by subtracting *e* we ensure that the mean phase over the inter-cellular domain is precisely the specified PFC parameter $\phi_{0}$ regardless of individual variation in values produced by each instance of the Gaussian random field.

We rescale the dimensions of an EM image with *x*-dimension length $L^{*}$ and horizontal grid spacing $h^{*}=L^{*}/M$ (for some *M*) to dimensionless length $L=2\pi L^{*}/l^{*}$ (as above) and grid spacing $h=\left(2\pi L^{*}\right)/\left(l^{*}M\right)$. Simulations were run in batches on the University of Manchester Computational Shared Facility. We use the DrWatson.jl package to manage results produced by testing the parameter space for these simulations (67).

## Appendix C: Image processing to define solution domains

To find a domain in which to solve the PFC equation, we started from cross-section EM images of embryonic tail tendon with x×y resolution M×N (Fig. 5 *a*). These images were converted to gray scale and binarized to a grid of black and white pixels. Binarized images were segmented to identify structures within the image using the Julia ImageSegmentation.jl package (Fig. 5 *b*). We identified the inter-cellular and intra-cellular spaces by filtering segments by size to leave only the two largest segments. We converted these segments to a matrix with values of 1 for all pixels in the inter-cellular space and 0 for all other pixels (Fig. 5 *c*).

Having created a binarized mask, we set diffusivity values for the PFC solution. We solved Eq. 1a on a periodic domain but restricted PFC evolution to the inter-cellular space by setting diffusivity to 1 in the inter-cellular space and 0 elsewhere. In practice, this means that diffusivity values were stored as a diagonal matrix *α* of dimension 2NM×2NM, where each diagonal component corresponds to the diffusivity of an edge connecting pixels in the binarized mask. If the value of either pixel is 0, then the diffusivity is 0; if both have a value of 1, then the corresponding diffusivity is 1 (Fig. 5 *d*).

## Appendix D: Parameters

Parameters used in our simulations are outlined in Table 1. A phase space illustrating the impact of varying *r* and $\phi_{0}$ on the patterns emerging at long times is given in Fig. 6. Larger $\phi_{0}$ and smaller *r* promote the appearance of patches in the domain that are not occupied by fibrils. The array of images indicates how the randomness of the initial conditions leads to variability in the patterns of fibrils that emerge and in the shapes of the fibril-free patches.

## Appendix E: Image analysis

To provide a more refined assessment of fibril patterns, we identified the locations of defects in an otherwise hexagonal array of fibrils as follows. Simulation results were processed by binarizing simulation results with a threshold of ϕ=0.5. These binarized results were segmented, again using the Julia ImageSegmentation.jl library. Fibril segments were obtained by filtering segments by pixel count, and the centroid position of each fibril was found by calculating the center of mass of each segment’s pixels. These centroids were analyzed in the same manner as fibril locations found manually in EM images.

For both EM images and simulation results, we performed a Delaunay triangulation over the set of centroid locations. We found a concave hull for the set of points using the Julia ConcaveHull.jl package. Fibrils within the concave hull set were considered to be peripheral fibrils and were excluded from subsequent analysis. We also excluded fibrils next to voids inside the lattice by calculating the mean area of internal Voronoi cells and excluding those with area greater than 1.3 times the mean from defect analysis. Using the Delaunay triangulation, we then determined fibril neighbor counts, distances between nearest neighbors, and lattice defect patterns (identifying internal fibrils that do not have six neighbors). The defect patterns of the simulations shown in Fig. 6 are shown in Fig. 7.

## Appendix F: Electron micrograph defects

Thirty examples of fibril patterns in EM images were analyzed to show defect patterns using the approach described in Appendix E, as shown in Fig. 8. The distributions of neighbor counts and the proportion of fibrils identified as defects are shown in Table 2. Representative simulations run in each of the 30 domains are shown in Fig. 9.

## Appendix G: MS data processing and analysis

Raw MS data were processed using MaxQuant (v.2.0.3.0) (68) against the mouse proteome downloaded from UniProt (August 2022) (69). Variable modifications were set as oxidation of methionine, oxidation of proline, and protein N-terminal acetylation, with a fixed modification as carbamidomethylation of cysteine. The false discovery rate (FDR) was set as 0.01 at both PSM and protein level. Tolerances were set at 20 and 4.5 ppm for the precursor in both first and main searches, with a 20-ppm tolerance set for tandem MS. The option “match between runs” was also selected. Known contaminants were removed.

Processed MS data were analyzed in R (v.4.1.2) (70) using the R package MSqRob2 (v.1.20) (71). Peptides were normalized using quantile normalization, and protein roll-up was performed using robust summarization. Time points were compared to the first (E12.5) time point, with significantly changing proteins taken at an FDR of 0.05. Heatmaps of relative change in protein abundance over time are shown in Fig. 10. Corresponding time-averaged absolute protein abundance across all five time points is shown in Fig. 11 *a*; a heatmap of absolute protein abundance is shown in Fig. 11 *b*.

## Supporting material

Data S1. Quantified protein intensities across time series annotated with Matrisome designations

## References

[1] Ricard-Blum S. (2011). The Collagen Family. CSH Perspect. Biol..

[2] Malfait F., Castori M., Byers P.H. (2020). The Ehlers-Danlos Syndromes. Nat. Rev. Dis. Prim..

[3] Rauch F., Glorieux F.H. (2004). Osteogenesis imperfecta. Lancet.

[4] Revell C.K., Jensen O.E., Kadler K.E. (2021). Collagen fibril assembly: New approaches to unanswered questions. Matrix Biol..

[5] Starborg T., Kadler K.E. (2015). Serial Block Face-Scanning Electron Microscopy: A Tool for Studying Embryonic Development at the Cell-Matrix Interface: SBF-SEM in developmental biology. Birth Defects Res. C.

[6] Musiime M., Chang J., Gullberg D. (2021). Collagen assembly at the cell surface: dogmas revisited. Cells.

[7] Canty E.G., Kadler K.E. (2005). Procollagen trafficking, processing and fibrillogenesis. J. Cell Sci..

[8] Graham H.K., Holmes D.F., Kadler K.E. (2000). Identification of Collagen Fibril Fusion during Vertebrate Tendon Morphogenesis. The Process Relies on Unipolar Fibrils and Is Regulated by Collagen-Proteoglycan Interaction. J. Mol. Biol..

[9] Canty E.G., Lu Y., Kadler K.E. (2004). Coalignment of plasma membrane channels and protrusions (fibripositors) specifies the parallelism of tendon. J. Cell Biol..

[10] Anderson P.W. (1994).

[11] Herrera J.A., Mallikarjun V., Swift J. (2020). Laser Capture Microdissection Coupled Mass Spectrometry (LCM-MS) for Spatially Resolved Analysis of Formalin-Fixed and Stained Human Lung Tissues. Clin. Proteonomics.

[12] van Huizen N.A., Ijzermans J.N.M., Luider T.M. (2020). Collagen Analysis with Mass Spectrometry. Mass Spectrom. Rev..

[13] Thiele U., Archer A.J., Knobloch E. (2013). Localized States in the Conserved Swift-Hohenberg Equation with Cubic Nonlinearity. Phys. Rev. E.

[14] Moelans N., Blanpain B., Wollants P. (2008). An Introduction to Phase-Field Modeling of Microstructure Evolution. Calphad.

[15] Archer A.J., Robbins M.J., Knobloch E. (2012). Solidification Fronts in Supercooled Liquids: How Rapid Fronts Can Lead to Disordered Glassy Solids. Phys. Rev. E.

[16] Chen L.-Q. (2002). Phase-Field Models for Microstructure Evolution. Annu. Rev. Mater. Res..

[17] Gránásy L., Tegze G., Pusztai T. (2011). Phase-field crystal modelling of crystal nucleation, heteroepitaxy and patterning. Philos. Mag. A.

[18] van Teeffelen S., Backofen R., Löwen H. (2009). Derivation of the Phase-Field-Crystal Model for Colloidal Solidification. Phys. Rev. E.

[19] Cameron S., Kreplak L., Rutenberg A.D. (2020). Phase-Field Collagen Fibrils: Coupling Chirality and Density Modulations. Phys. Rev. Res..

[20] Leighton M.P., Kreplak L., Rutenberg A.D. (2021). Non-Equilibrium Growth and Twist of Cross-Linked Collagen Fibrils. Soft Matter.

[21] Emmerich H., Löwen H., Gránásy L. (2012). Phase-Field-Crystal Models for Condensed Matter Dynamics on Atomic Length and Diffusive Time Scales: An Overview. Adv. Phys. X..

[22] Asadi E., Asle Zaeem M. (2015). A Review of Quantitative Phase-Field Crystal Modeling of Solid–Liquid Structures. J. Occup. Med..

[23] Giraud-Guille M.-M., Besseau L., Martin R. (2003). Liquid crystalline assemblies of collagen in bone and in vitro systems. J. Biomech..

[24] Saeidi N., Karmelek K.P., Ruberti J.W. (2012). Molecular Crowding of Collagen: A Pathway to Produce Highly-Organized Collagenous Structures. Biomaterials.

[25] Abhilash A.S., Baker B.M., Shenoy V.B. (2014). Remodeling of fibrous extracellular matrices by contractile cells: predictions from discrete fiber network simulations. Biophys. J..

[26] Grekas G., Proestaki M., Ravichandran G. (2021). Cells exploit a phase transition to mechanically remodel the fibrous extracellular matrix. J. R. Soc. Interface.

[27] Lin J., Shi Y., Zhang C. (2020). Mechanical Roles in Formation of Oriented Collagen Fibers. Tissue Eng. Part B.

[28] Bueno J., Starodumov I., Alexandrov D. (2016). Three Dimensional Structures Predicted by the Modified Phase Field Crystal Equation. Comput. Mater. Sci..

[29] Prieler R., Hubert J., Emmerich H. (2009). An Anisotropic Phase-Field Crystal Model for Heterogeneous Nucleation of Ellipsoidal Colloids. J. Phys. Condens. Matter.

[30] Zaeem M.A., Nouranian S., Horstemeyer M.F. (2012). Proceedings of the 2012 AIChE Annual Meeting.

[31] Rutenberg A.D., Brown A.I., Kreplak L. (2016). Uniform Spatial Distribution of Collagen Fibril Radii within Tendon Implies Local Activation of pC-collagen at Individual Fibrils. Phys. Biol..

[32] Lord G.J., Powell C.E., Shardlow T. (2014).

[33] Glasner K., Orizaga S. (2016). Improving the Accuracy of Convexity Splitting Methods for Gradient Flow Equations. J. Comput. Phys..

[34] Bezanson J., Edelman A., Shah V.B. (2017). Julia: A Fresh Approach to Numerical Computing. SIAM Rev..

[35] Rackauckas C., Nie Q. (2017). DifferentialEquations.jl – a performant and feature-rich ecosystem for solving differential equations in Julia. J. Open Res. Software.

[36] Aurenhammer F. (1991). Voronoi diagrams—a survey of a fundamental geometric data structure. ACM Comput. Surv..

[37] Jiménez F.L., Stoop N., Reis P.M. (2016). Curvature-Controlled Defect Localization in Elastic Surface Crystals. Phys. Rev. Lett..

[38] Herrera J.A., Dingle L., Schwartz M.A. (2022). The UIP/IPF Fibroblastic Focus Is a Collagen Biosynthesis Factory Embedded in a Distinct Extracellular Matrix. JCI Insight.

[39] Kohrs R.T., Zhao C., Amadio P.C. (2011). Tendon Fascicle Gliding in Wild Type, Heterozygous, and Lubricin Knockout Mice: Lubricin Affects Tendon Fascicle Gliding. J. Orthop. Res..

[40] Herrera J.A., Dingle L.A., Thornton D.J. (2022). The UIP honeycomb airway cells are the site of mucin biogenesis with deranged cilia. bioRxiv.

[41] Irvine W.T.M., Vitelli V., Chaikin P.M. (2010). Pleats in crystals on curved surfaces. Nature.

[42] Danisch S., Krumbiegel J. (2021). Makie.jl: Flexible high-performance data visualization for Julia. J. Open Source Softw..

[43] Starborg T., Kalson N.S., Kadler K.E. (2013). Using Transmission Electron Microscopy and 3View to Determine Collagen Fibril Size and Three-Dimensional Organization. Nat. Protoc..

[44] Siadat S.M., Silverman A.A., Ruberti J.W. (2021). Measuring collagen fibril diameter with differential interference contrast microscopy. J. Struct. Biol..

[45] Naba A., Clauser K.R., Hynes R.O. (2012). The Matrisome: In Silico Definition and In Vivo Characterization by Proteomics of Normal and Tumor Extracellular Matrices. Mol. Cell. Proteomics.

[46] Wenstrup R.J., Smith S.M., Birk D.E. (2011). Regulation of Collagen Fibril Nucleation and Initial Fibril Assembly Involves Coordinate Interactions with Collagens V and XI in Developing Tendon. J. Biol. Chem..

[47] Zhang G., Ezura Y., Birk D.E. (2006). Decorin Regulates Assembly of Collagen Fibrils and Acquisition of Biomechanical Properties during Tendon Development. J. Cell. Biochem..

[48] Ezura Y., Chakravarti S., Birk D.E. (2000). Differential Expression of Lumican and Fibromodulin Regulate Collagen Fibrillogenesis in Developing Mouse Tendons. J. Cell Biol..

[49] Ito S., Nagata K. (2017). Biology of Hsp47 (Serpin H1), a Collagen-Specific Molecular Chaperone. Semin. Cell Dev. Biol..

[50] Park N.R., Shetye S.S., Joeng K.S. (2021). Reticulocalbin 3 Is Involved in Postnatal Tendon Development by Regulating Collagen Fibrillogenesis and Cellular Maturation. Sci. Rep..

[51] Barnes A.M., Chang W., Marini J.C. (2006). Deficiency of Cartilage-Associated Protein in Recessive Lethal Osteogenesis Imperfecta. N. Engl. J. Med..

[52] Pihlajaniemi T., Myllylä R., Kivirikko K.I. (1991). Prolyl 4-Hydroxylase and Its Role in Collagen Synthesis. J. Hepatol..

[53] Schegg B., Hülsmeier A.J., Hennet T. (2009). Core Glycosylation of Collagen Is Initiated by Two β(1-O)Galactosyltransferases. Mol. Cell Biol..

[54] Rezaei N., Lyons A., Forde N.R. (2018). Environmentally controlled curvature of single collagen proteins. Biophys. J..

[55] Claire K., Pecora R. (1997). Translational and Rotational Dynamics of Collagen in Dilute Solution. J. Phys. Chem. B.

[56] Belamie E., Mosser G., Giraud-Guille M.-M. (2006). Possible transient liquid crystal phase during the laying out of connective tissues: α-chitin and collagen as models. J. Phys. Condens. Matter.

[57] Giraud-Guille M.M., Mosser G., Belamie E. (2008). Liquid crystallinity in collagen systems in vitro and in vivo. Curr. Op. Coll. Interface Sci..

[58] Forgacs G., Newman S.A., Sackmann E. (2003). Assembly of collagen matrices as a phase transition revealed by structural and rheologic studies. Biophys. J..

[59] Bard J.B., Chapman J.A. (1968). Polymorphism in Collagen Fibrils precipitated at Low pH. Nature.

[60] Kadler K.E., Hojima Y., Prockop D.J. (1987). Assembly of collagen fibrils de novo by cleavage of the type I pC-collagen with procollagen C-proteinase. Assay of critical concentration demonstrates that collagen self-assembly is a classical example of an entropy-driven process. J. Biol. Chem..

[61] Hulmes D.J.S. (2002). Building collagen molecules, fibrils, and suprafibrillar structures. J. Struct. Biol..

[62] Shayegan M., Forde N.R. (2013). Microrheological characterization of collagen systems: from molecular solutions to fibrillar gels. PLoS One.

[63] Renner-Rao M., Jehle F., Harrington M.J. (2022). Mussels Fabricate Porous Glues via Multiphase Liquid–Liquid Phase Separation of Multiprotein Condensates. ACS Nano.

[64] Grason G.M. (2016). Perspective: Geometrically frustrated assemblies. J. Chem. Phys..

[65] Perez-Riverol Y., Bai J., Bandla C., izcaíno J.A. (2022). The PRIDE database resources in 2022: a hub for mass spectrometry-based proteomics evidences. Nucleic Acids Res.

[66] Revell C.K. (2023). PhaseFieldCrystal.jl. https://github.com/chris-revell/PhaseFieldCrystal.

[67] Datseris G., Isensee J., Gál T. (2020). DrWatson: The Perfect Sidekick for Your Scientific Inquiries. J. Open Source Softw..

[68] Tyanova S., Temu T., Cox J. (2016). The MaxQuant computational platform for mass spectrometry-based shotgun proteomics. Nat. Protoc..

[69] UniProt Consortium (2021). UniProt: the universal protein knowledgebase in 2021. Nucleic Acids Res..

[70] R Core Team (2013).

[71] Goeminne L.J.E., Gevaert K., Clement L. (2018). Experimental design and data-analysis in label-free quantitative LC/MS proteomics: A tutorial with MSqRob. J. Proteonomics.

